# Fermentative production of the unnatural amino acid l-2-aminobutyric acid based on metabolic engineering

**DOI:** 10.1186/s12934-019-1095-z

**Published:** 2019-02-28

**Authors:** Jian-Miao Xu, Jian-Qiang Li, Bo Zhang, Zhi-Qiang Liu, Yu-Guo Zheng

**Affiliations:** Key Laboratory of Bioorganic Synthesis of Zhejiang Province, College of Biotechnology and Bioengineering, Zhejiang University of Technology, Hangzhou, 310014 China

**Keywords:** l-2-Aminobutyric acid, l-Threonine deaminase, l-Leucine dehydrogenase, Metabolic engineering, Fed-batch fermentation

## Abstract

**Background:**

l-2-aminobutyric acid (l-ABA) is an unnatural amino acid that is a key intermediate for the synthesis of several important pharmaceuticals. To make the biosynthesis of l-ABA environmental friendly and more suitable for the industrial-scale production. We expand the nature metabolic network of *Escherichia coli* using metabolic engineering approach for the production of l-ABA.

**Results:**

In this study, *Escherichia coli* THR strain with a modified pathway for threonine-hyperproduction was engineered via deletion of the *rhtA* gene from the chromosome. To redirect carbon flux from 2-ketobutyrate (2-KB) to l-ABA, the *ilvIH* gene was deleted to block the l-isoleucine pathway. Furthermore, the *ilvA* gene from *Escherichia coli* W3110 and the *leuDH* gene from *Thermoactinomyces intermedius* were amplified and co-overexpressed. The promoter was altered to regulate the expression strength of ilvA* and *leuDH*. The final engineered strain *E. coli* THR Δ*rhtA*Δ*ilvIH*/Gap-ilvA*-Pbs-*leuDH* was able to produce 9.33 g/L of l-ABA with a yield of 0.19 g/L/h by fed-batch fermentation in a 5 L bioreactor.

**Conclusions:**

This novel metabolically tailored strain offers a promising approach to fulfill industrial requirements for production of l-ABA.

**Electronic supplementary material:**

The online version of this article (10.1186/s12934-019-1095-z) contains supplementary material, which is available to authorized users.

## Background

l-2-aminobutyric acid (l-ABA), a nonnatural amino acid, has been used as a precursor for synthesis of many chiral drugs, such as anti-epileptic Levetiracetam, anti-tuberculotic Ethambutol and Brivaracetam [1–3]. (*S*)-2-amino butanol, a key intermediate of Ethambutol, can be synthesized by esterification and hydrogenation reduction of l-ABA [4]. With the increasing market demand for l-ABA in both pharmaceutical and chemical industries in recent years, the preparation of optically pure l-ABA with high efficacy has attracted much attention.

Currently, the preparation of l-ABA is mainly achieved by chemical synthesis or enzymatic conversion. In chemical methods, synthesis of l-ABA has been extensively reported including ammonolysis of α-halogen acid [5], reduction reaction [6], ammoniation hydrolysis reaction and butanone acid reduction [7]. However, the obvious disadvantages of chemical synthesis, such as poor selectivity, harsh reaction conditions, various byproducts, and the difficulty in separation and purification [8–10], limited its development. Enzymatic synthesis of l-ABA is emerged since biotransformation and bio-refining for the green production of chemicals have been attracting the increasing attention due to serious concerns about climate change and environmental problems [11–13]. It is reported that l-ABA was synthesized in a transamination reaction from α-ketobutyric acid and l-aspartic acid as substrates using aromatic aminotransferase [14] or produced from α-ketobutyric acid and benzylamine using ω-aminotransferase [1]. l-ABA could also be produced from the reduction of α-keto acids with l-leucine dehydrogenase [15] or glutamate dehydrogenase [16]. But in the enzymatic routes, the presence of by-product α-keto acid decreases the overall yield and purity of l-ABA [2]. It is well known that, most of the natural l-amino acids can now be produced from glucose by microbial fermentation [17]. Notably, l-glutamate, l-lysine, and l-threonine are produced more than 2 million tons annually [18]. Therefore, microbial production of l-ABA from cheap and clean resources has gained much attention with the potential to overcome these problems.

*Escherichia coli* has been reported to produce many natural amino acids owing to its clear genetic background and facile genetic manipulation [17, 19]. At present, there are few reports about biosynthesis of the nonnatural amino acid l-ABA using microbial fermentation. We attempt to construct a novel engineered *E. coli* strain for production of the nonnatural amino acid, l-ABA directly from glucose by expanding its metabolic pathways.

In this study, a threonine producing strain *E. coli* THR was firstly constructed, then the metabolic pathway was expanded via specific genes overexpression for converting l-threonine to l-ABA. Further modification for improvement of l-ABA production included l-threonine reduction, l-isoleucine synthetic pathway blockage and promoter replacement. This work provides a novel approach for the industrial production of l-ABA by fermentation using the genetically engineered *E. coli* strain.

## Results

### Construction of engineered *E. coli* strain for l-threonine production

**Fig. 1:**
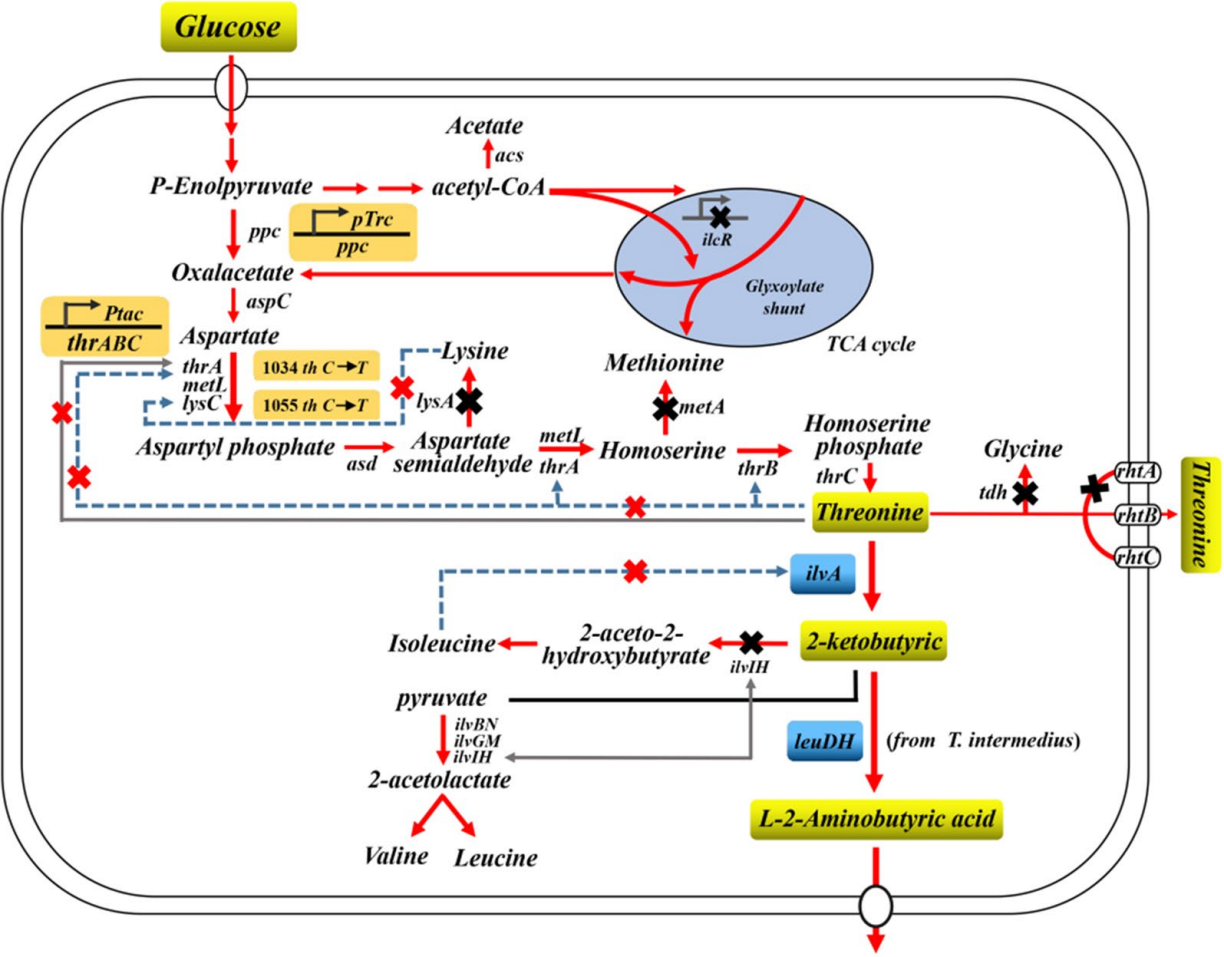
The overall metabolic engineering strategy employed for the construction of a genetically defined l-ABA producer. Central metabolic pathways that contribute to the biosynthesis of l-ABA together with competing pathways and regulatory circuits are shown. The orange shaded boxes represent mutations introduced into the genome. The black Xs indicate the deletion of genes and the red Xs indicate the removal of inhibition or repression. Dotted lines indicate the feedback inhibition. Gray lines indicate the transcriptional attenuation regulation. The red thick arrows indicate the increased flux or activity by directly overexpressing the corresponding genes

To expand metabolism for producing l-ABA, we designed a unique metabolic pathway in *E. coli*, where l-threonine was taken as a precursor for l-ABA synthesis (Fig. 1). In this work, *E. coli* THR strain (Table 1) was constructed for the overproduction of l-threonine. The feedback inhibitions of aspartokinase I and III encoded by *thrA* and *lysC* genes, respectively, were released [20]. The native promoter containing the transcriptional attenuator leader region of the *thrABC* operon was replaced with the *tac* promoter. The *metA*, *lysA*, *tdh* and *iclR* genes were deleted to make more precursors available for l-threonine formation. The native promoter of the *ppc* gene was replaced with the Trc promoter in the chromosome to increase the pool of oxaloacetate, a starting precursor of l-threonine biosynthesis [21]. As a result, the strain *E. coli* THR produced 12.45 g/L l-threonine from 50 g/L glucose in shake flask at 35 °C for 48 h in TPM medium, which was further used to construct an l-ABA producing strain.

**Table 1:** Characteristics and sources of bacterial strains used in this study

Strains	Characteristics	Sources
*E. coli* W3110	*Coli* Genetic Stock Center strain (CGSC) No. 4474	CGSC^a^
*E. coli* TOP10	F^−^ *mcrA* Δ(*mrr*-*hsdRMS*-*mcrBC*) φ*80lacZ*ΔM15 Δ*lacX74 deoR recA1 araD139* Δ(*ara*-*leu*)*7697galU galK rpsL endA1 nupG*	Invitrogen^b^
*E. coli* THR	W3110 (Δ*lacI*, *thrA*^C1034T^, *lysC*^C1055T^, P*thrABC*::P*tac*, Δ*lysA*, Δ*metA*, Δ*tdh*, Δ*iclR*, P*ppc*::PTrc)	This study
*E. coli* THR Δ*rhtA*	*E. coli* THR derivative, Δ*rhtA*	This study
*E. coli* THR Δ*rhtC*	*E. coli* THR derivative, Δ*rhtC*	This study
*E. coli* THR Δ*rhtA*Δ*rhtC*	*E. coli* THR derivative, Δ*rhtA*/Δ*rhtC*	This study
*E. coli* THR Δ*rhtA*Δ*ilvIH*	*E. coli* THR derivative, Δ*rhtA*/Δ*ilvIH*	This study

^a^Invitrogen, Crop., Carlsbad, CA

^b^Coli Genetic Stock Center

### Reprogramming of *ilvA* to redirect the carbon flux towards 2-KB

**Fig. 2:**
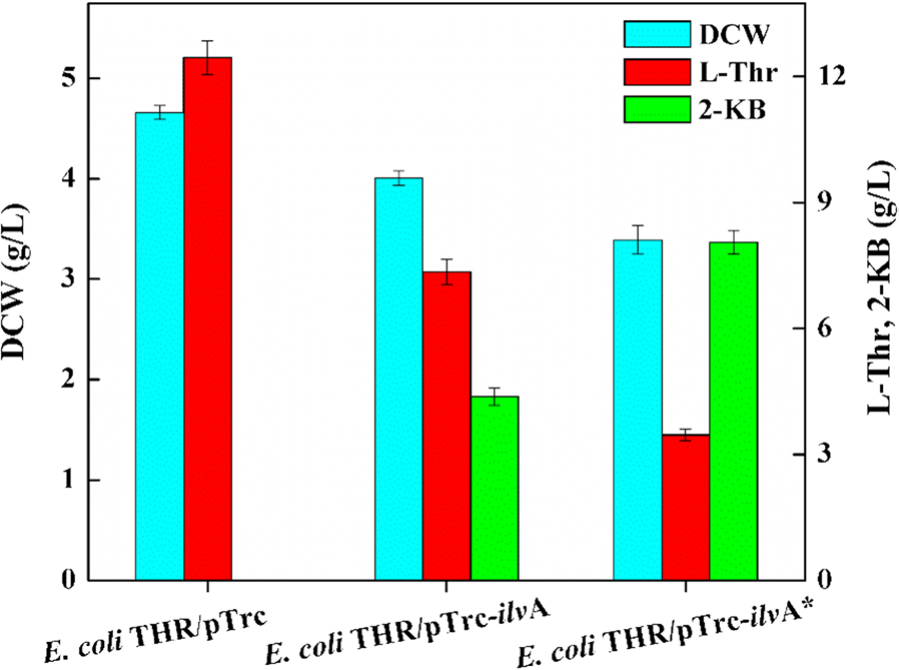
Effect of ilvA* overexpression on 2-KB titer

It is well known that l-threonine was catalyzed to form 2-KB by threonine dehydratase encoded by *ilvA* in *E. coli*. In this study, an *ilvA* overexpression strain was constructed, which could produce 4.38 g/L 2-KB and 7.35 g/L l-threonine (Fig. 2). The high accumulation of l-threonine intracellularly indicated that the catabolic enzyme *ilvA* is not active enough to fully convert l-threonine into 2-KB. The feedback inhibition of *ilvA* by l-isoleucine was then considered as the main factor to hinder the further improvement of the 2-KB titer [16].

On the basis of previous studies, feedback inhibition of *ilvA* could be removed by replacing the 1054 th T with G, 1055 th T with C, 1084 th C with T, 1085 th G with T and 1086 th T with C (F352A, R362F) using site-directed mutagenesis to obtain pTrc-ilvA* [22]. The 2-KB titer of *E. coli* THR/pTrc-ilvA* increased by 83.8% which was up to 8.05 g/L, the value of which was nearly 2 times of that from the pTrc-*ilvA* strain (4.38 g/L 2-KB), and the concentration of remaining l-threonine from 7.35 g/L decrease to 3.47 g/L (Fig. 2). These results suggested that the removal of l-isoleucine which is the feedback inhibition of the *ilvA* gene could increase both the activity and resistance to l-isoleucine inhibition as compared to that of the wild-type strain and drive the carbon flux from l-threonine to 2-KB [22].

### Enzyme selection for converting 2-KB to l-ABA

**Fig. 3:**
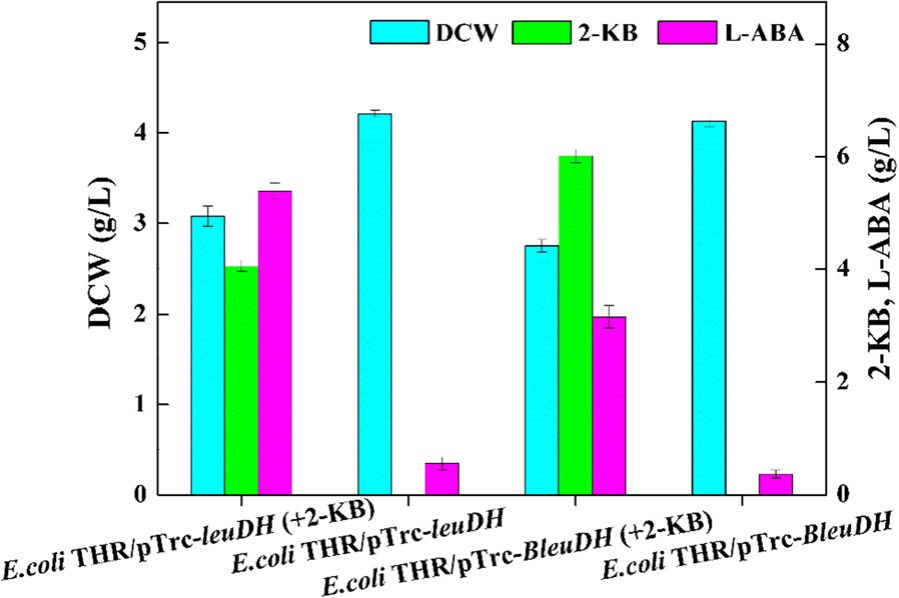
Effects of *leuDH* and *BleuDH* overexpression on l-ABA titer

In order to gain an appropriate dehydrogenase for 2-KB production, two different sources of dehydrogenase, including *leuDH* from *T. intermedius* [23] and *BleuDH* from *Bacillus cereus* [24] were tested. Plasmids pTrc-*leuDH* and pTrc-*BleuDH* were constructed and introduced into *E. coli* THR. Results showed that the accumulation of l-ABA reached to 5.39 g/L and 3.16 g/L in *E. coli* THR/pTrc-*leuDH* and *E. coli* THR/pTrc-*BleuDH*, respectively, which were cultivated in TPM medium with additional feeding of 10 g/L 2-KB (Fig. 3). These results demonstrated that the dehydrogenase *leuDH* from *T. intermedius* displayed a higher specific activity in *E. coli* THR than that of *Bleu*DH from *Bacillus cereus*.

### Modification of bypass pathway to further improve the l-ABA titer

**Fig. 4:**
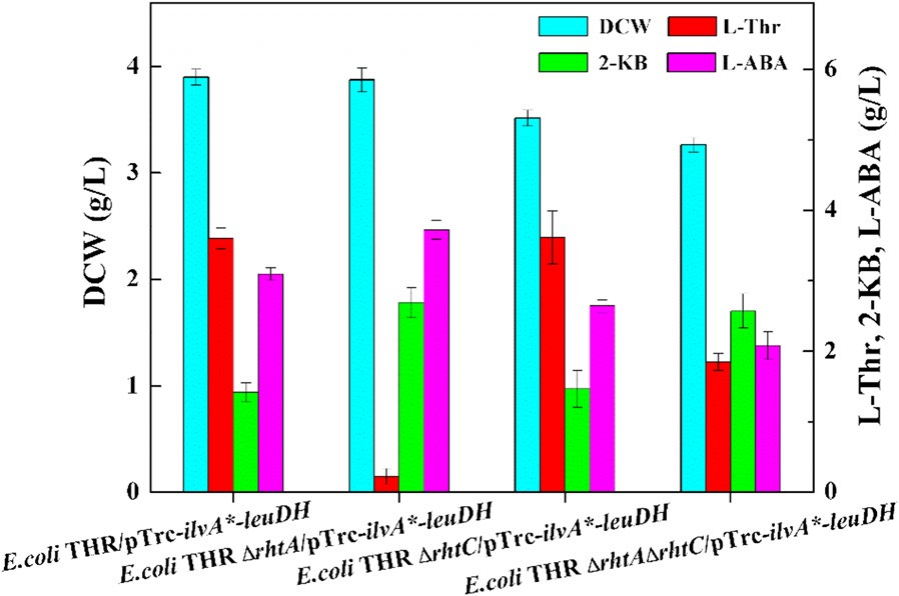
Effect of *rht*A or *rht*C deletion on l-ABA titer

Based on the fermentation results from ilvA* and *leuDH* expression separately in *E. coli* THR, the co-overexpression strain *E. coli* THR/pTrc-*ilv*A*-*leuDH* was constructed, which could produce 3.09 g/L l-ABA from 50 g/L glucose. However, there was still 3.47 g/L l-threonine remained in the fermentation medium (Fig. 4). In *E. coli*, *rhtA* and *rhtC* are known to be involved in l-threonine efflux [25, 26]. When the *rhtA* gene was overexpressed on multicopy plasmids, the expression of the *rhtA* gene enhanced about tenfold [27]. The *rhtC* gene was induced to protect cells from toxic effects of intracellular l-threonine accumulation by exporting l-threonine out of the cell. It is reported that overexpression of the *rhtC* gene could increase the production of l-threonine which is 50.2% higher than that without *rhtC* amplification [21].

With the purpose of decreasing the l-threonine export capacity, the gene *rhtA* and *rhtC* were deleted from the *E. coli* THR strain chromosome, resulting in three strains *E. coli* THR Δ*rhtA*, *E. coli* THR Δ*rhtC* and *E. coli* THR Δ*rhtA*Δ*rhtC*. Then the recombinant plasmid pTrc-ilvA*-*leuDH* was respectively transformed into three strains to test the l-ABA production. The result of strain *E. coli* THR Δ*rhtA*/pTrc-ilvA*-*leuDH* displayed higher concentration of l-ABA than the other two strains (Fig. 4). The deletion of *rhtA* in the chromosome led to the increase of l-ABA concentration from 3.09 g/L to 3.72 g/L and the remaining l-threonine decrease from 3.47 g/L to 0.22 g/L, which indicated that the modification of the l-threonine transport pathway is an efficient strategy for l-ABA improvement.

**Fig. 5:**
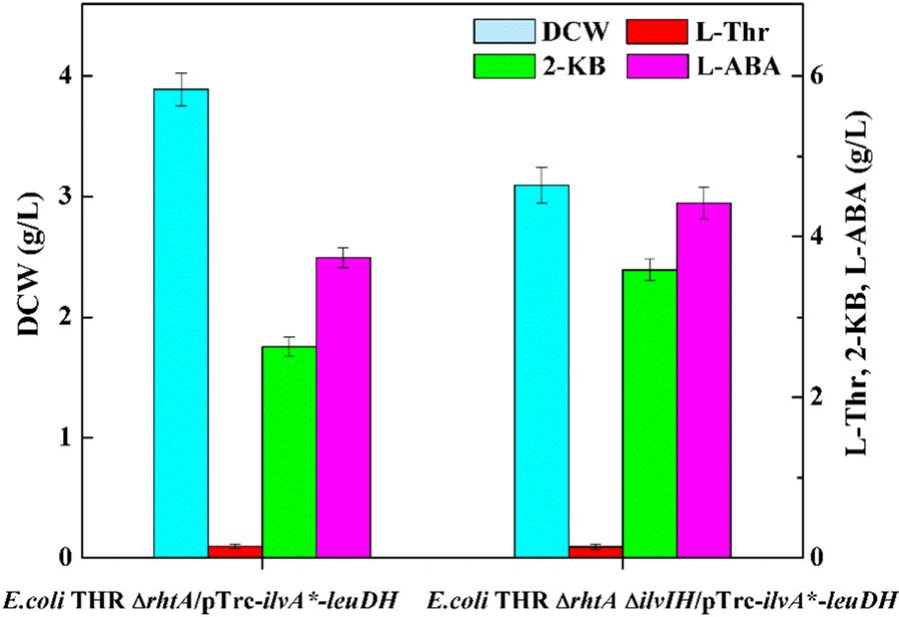
Effect of *ilvIH* deletion on l-ABA titer

In addition, three acetohydroxy acid synthase (AHAS) isoenzymes that *E. coli* possessed, which show different biochemical properties and regulation mechanisms, play important roles in the biosynthesis of l-isoleucine. Among them, AHAS III, encoded by *ilvIH*, exhibites a much higher affinity for 2-KB [28] and AHAS I, encoded by *ilvBN*, displays a higher affinity for pyruvate than 2-KB [28]. However, AHAS II, encoded by *ilvGM*, is not expressed due to the frameshift mutation of *ilvG* in *E. coli* [29]. Thus, only the gene *ilvIH* from the *E. coli* THR Δ*rht*A chromosome was knocked out to reduce the metabolic flux from 2-KB to l-isoleucine, the resulting strain *E. coli* THR Δ*rht*AΔ*ilvIH/*pTrc-ilvA*-*leuDH* was capable of producing 4.42 g/L l-ABA (Fig. 5).

### Regulation of the expression strength of ilvA* and *leuDH* via promoter alteration

**Fig. 6:**
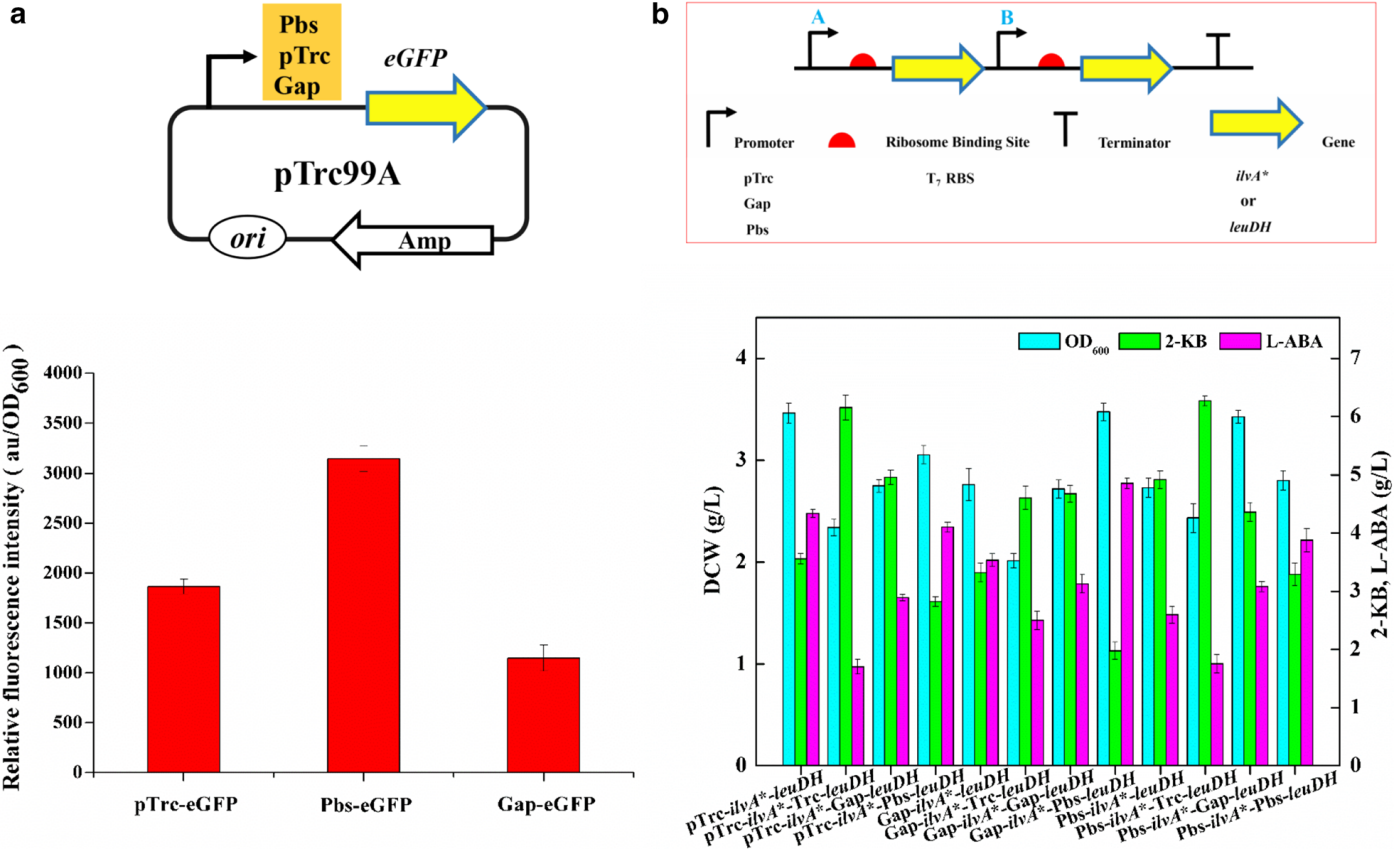
Regulating the expression of ilvA* and *leuDH* for l-ABA titer. **a** Relative fluorescence intensity (au/OD_600_) of eGFP harbored in different promoters. **b** Effect of promoters with different strengths on l-ABA titer

It was observed that 3.56 g/L 2-KB was remained in flask culture of *E. coli* THR Δ*rht*AΔ*ilvIH/*pTrc-ilvA*-*leuDH*. We reasoned that the uncoordinated expression of ilvA* and *leuDH* might account for the extracellular accumulation of 2-KB. Therefore, it is necessary to regulate the expression of ilvA* and *leuDH* via promoter alteration. We employed different promoters with different strengths, including Pbs [30], Trc and Gap from *E. coli* BL21 (DE3), to regulate the expression of ilvA* and *leuDH* based on plasmid pTrc-ilvA*-*leuDH*. To investigate the strengths of different promoters, enhanced green fluorescent protein (eGFP) was introduced and used as a reporter. As a result, eGFP was successfully expressed under the control of three promoters, respectively in *E. coli* THR Δ*rht*AΔ*ilvIH* and the relative fluorescence intensity (au/OD600) from strong to weak was Pbs-eGFP, Trc-eGFP and Gap-eGFP (Fig. 6a). Twelve recombinant plasmids were constructed when three promoters were inserted or replaced before the ilvA* or *leuDH*, respectively (Table 2). The results showed that 4.86 g/L of l-ABA was produced by *E. coli* THR Δ*rht*AΔ*ilvIH/*Gap-ilvA*-Pbs-*leuDH* (Fig. 6b), which was the strain with the highest yield of l-ABA compared with other strains constructed in this study. Moreover, the accumulation of 2-KB was decreased to 1.98 g/L, whereas the cell growth did not change remarkably.

**Table 2:** Descriptions and sources of plasmids used in this study

Plasmids	Description	Sources
pTrc99a	AmpR, pBR322-origin, trc promoter, 4.2 kb	[35, 38]
pCas	Kan^R^, Cas9 nuclease expression plasmid, temperature-sensitive origin	[39, 40]
pTrc-ilvA*	AmpR, ilvA* cloned in the *sac *I and *BamH* I site of pTrc99A	This study
Gap-ilvA*	Amp^R^, replacement of Trc promoter in pTrc-ilvA* with the Gap promoter	This study
Pbs-ilvA*	Amp^R^, replacement of Trc promoter in pTrc-ilvA* with the Pbs promoter	This study
pTrc-ilvA* -*BleuDH*	Amp^R^, *BleuDH* cloned in the *Xba *I and *Hind *III site of pTrc-ilvA*	This study
pTrc-ilvA*-*leuDH*	AmpR, *leuDH* cloned in the *Xba *I and *Hind* III site of pTrc-ilvA*	This study
pTrc-ilvA*-Trc-*leuDH*	Amp^R^, *leuDH* carrying a Trc promoter and RBS cloned in the *Xba *I and *Hind* III site of pTrc-ilvA*	This study
pTrc-ilvA*-Gap-*leuDH*	Amp^R^, *leuDH* carrying a Gap promoter and RBS cloned in the *Xba *I and *Hind* III site of pTrc-ilvA*	This study
pTrc-*ilv*A*-Pbs-*leuDH*	Amp^R^, *leuDH* carrying a Pbs promoter and RBS cloned in the *Xba *I and *Hind* III site of pTrc-ilvA*	This study
Gap-ilvA*-*leuDH*	Amp^R^, replacement of Trc promoter in pTrc-*ilv*A*-*leuDH* with the Gap promoter	This study
Gap-ilvA*-Trc-*leuDH*	Amp^R^, *leuDH* carrying a Trc promoter and RBS cloned in the *Xba *I and *Hind* III site of Gap-ilvA*	This study
Gap-ilvA*-Gap-*leuDH*	Amp^R^, *leuDH* carrying a Gap promoter and RBS cloned in the *Xba *I and *Hind* III site of Gap-ilvA*	This study
Gap-ilvA*-Pbs-*leuDH*	Amp^R^, *leuDH* carrying a Pbs promoter and RBS cloned in the *Xba *I and *Hind* III site of Gap-ilvA*	This study
Pbs-ilvA*-*leuDH*	Amp^R^, replacement of Trc promoter in pTrc-ilvA*-*leuDH* with the Pbs promoter	This study
Pbs-ilvA*-Trc-*leuDH*	Amp^R^, *leuDH* carrying a Trc promoter and RBS cloned in the *Xba *I and *Hind* III site of Pbs-ilvA*	This study
Pbs-ilvA*-Gap-*leuDH*	Amp^R^, *leuDH* carrying a Gap promoter and RBS cloned in the *Xba *I and *Hind* III site of Pbs-ilvA*	This study
Pbs-ilvA*-Pbs-*leuDH*	Amp^R^, *leuDH* carrying a Pbs promoter and RBS cloned in the *Xba *I and *Hind* III site of pbs-ilvA*	This study
pTarget-X	A plasmid used to transcript sgRNA targeting the particular gene X in genome. X refers to the amino acid biosynthetic pathways	This study

*Sp* spectinomycin, *Kan* kanamycin, *Amp* ampicillin, *R* resistance

### Fed-batch fermentation for l-ABA production

**Fig. 7:**
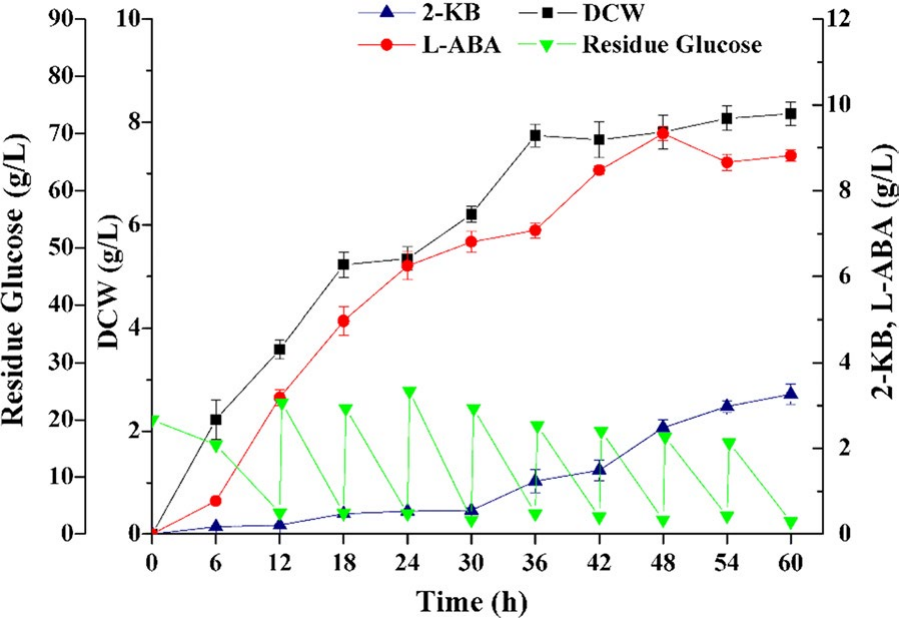
Fermentation process profiles of *E. coli* THR Δ*rhtA*Δ*ilvIH*/Gap-ilvA*-Pbs-*leuDH* showing DCW (filled squares), residual glucose (filled inverse triangle), 2-KB titer (filled upright triangle) and l-ABA titer (filled circles)

As the platform strains for the production of l-ABA through rational metabolic engineering was successfully constructed, fed-batch fermentation was performed to evaluate the potential of the engineered strain *E. coli* THR Δ*rhtA*Δ*ilvIH/*Gap-ilvA*-Pbs-*leuDH*. During the fermentation, the initially-fed glucose was exhausted at approximately 12 h. When the glucose concentration in the broth was less than 5 g/L, feed medium was injected into the broth to raise the residual glucose concentration to around 20 g/L. Production of l-ABA presented a cell growth-dependent profile in the first 36 h of the fermentation. When the growth of the cells entered the stationary phase, l-ABA was continuously accumulated, but at a lower rate than that in the exponential phase, followed by a slight decrease in the titer after 48 h of the fermentation (Fig. 7). As a result, 9.33 g/L of l-ABA was produced from glucose in a total 60 h of fed-batch fermentation, representing a 1.92-fold increase compared to the titer achieved in shake flask. Meanwhile, 2-KB was detected as the main byproduct during the fermentation in the 5 L bioreactor with the titer of 3.27 g/L. Another byproduct l-threonine was barely detected.

## Discussion

In this study, we have developed a novel biosynthesis process for l-ABA production directly from glucose by fermentation using the genetically engineered *E. coli* strain. The results confirmed that the high expression level of *leuDH* and ilvA* successfully expanded the biosynthetic pathway, resulting in 3.60 g/L l-threonine, 1.42 g/L 2-KB and 3.09 g/L l-ABA in shake flask. Comparing to the traditional enzyme catalytic production method, this synthetic process possesses the advantages of simplicity in the production process and the production costs-savings, which represents an attractive approach for the production of various valuable nonnatural products from inexpensive renewable resources.

To avoid the excessive accumulation of extracellular l-threonine, a partial inactivation of the l-threonine export system was employed. In accordance with the previous reports [21, 27], *rhtA* and *rhtC*, which encoded strong l-threonine exporter, were deleted from the *E. coli* THR strain chromosome to inactivate the l-threonine export system. Our result indicated that it was better to knock out the *rhtA* gene alone rather than to knock out the *rhtC* gene alone or both genes at the same time regarding to the l-ABA production. The *rht*A-deletion strain *E. coli* THR Δ*rht*A/pTrc-ilvA*-*leuDH* showed an l-ABA production enhancement by 20.4% in comparison with that of *E. coli* THR/pTrc-ilvA*-*leuDH.* The results further indicated that the reasonable modification of l-threonine transport system was beneficial to the accumulation of l-ABA.

As competitive branches, the disruption of l-isoleucine biosynthetic pathways should be contributed to the improvement of l-ABA production [31]. Block of the l-isoleucine metabolic pathway by disrupting *ilvIH* made the titer of l-ABA in *E. coli* THR Δ*rhtA*Δ*ilvIH*/pTrc-ilvA*-*leuDH* increased by 1.19-fold compared to that in *E. coli* THR Δ*rhtA*/pTrc-ilvA*-*leuDH*, but the cell growth decreased. It is concluded that *ilvIH* disruption allowed the diversion of 2-KB flowed to l-isoleucine to l-ABA, and therefore improved the titer of l-ABA, but caused accumulation of 2-KB to the value of 3.56 g/L, which led to a reduction in cell growth [31]. Interestingly, the l-ABA titer was accumulated to 4.86 g/L and 2-KB decreased to 1.98 g/L in *E. coli* THR Δ*rhtA*Δ*ilvIH*/Gap-ilvA*-Pbs-*leuDH* without any obvious reduction of cell growth when promoters of different strengths including Pbs [30] and Gap from *E. coli* BL21 (DE3) were employed to regulate the expression of ilvA* and *leuDH*. These results indicated that the disruption of *ilvIH* drived more carbon flux to l-ABA and proper adjustments of attenuating the expression of ilvA* by a relatively weak promoter and enhancing the expression of *leuDH* by a strong promoter were also beneficial for the biosynthesis of l-ABA in this strain.

This work reported the development of a bacterial platform for enhanced production of a nonnatural amino acid l-ABA. The highest yield of l-ABA achieved by the engineered *E. coli* strain was 9.33 g/L via fed-batch fermentation, indicating a great potential for large-scale production. However, there are still some bottlenecks, including the intracellular l-ABA transfer, plasmid elimination, metabolic burden and high costs of fermentation due to the large amount requirement of glucose and high concentration of l-methionine, l-isoleucine and l-lysine, waiting to be conquered for the industrial production of l-ABA. Therefore, screening of relevant genes encoding l-ABA exporter and integrating the involved genes into the chromosome of *E. coli* THR will be carried out in the future. In addition, the fermentation medium and condition will be further optimized to reduce the production costs and increase the l-ABA titer.

## Conclusions

In this study, a novel strain *E. coli* THR Δ*rhtA*Δ*ilvIH*/Gap-ilvA*-Pbs-*leuDH* for l-ABA production was constructed through metabolic engineering. The *ilvA* gene from *E. coli* W3110 and the *leuDH* gene from *T. intermedius* were firstly co-expressed in *E. coli* THR, and 3.09 g/L of l-ABA was achieved. Then, the *rhtA* gene was disrupted to decrease the extracellular secretion of l-threonine and the titer of l-ABA was improved to 3.72 g/L. To block the catabolism from 2-KB to l-isoleucine, the *ilvIH* gene was disrupted and the l-ABA titer was increased by 18.8% compared to the parental strain. Furthermore, in order to obtain a better expression of *ilvA* and *leuDH*, different promoters were, respectively inserted or replaced before the two enzymes to regulate their expression, resulting in l-ABA accumulation up to 4.86 g/L. Finally, l-ABA titer of the optimal strain *E. coli* THR Δ*rhtA*Δ*ilvIH*/Gap-ilvA*-Pbs-*leuDH* reached to 9.33 g/L in fed-batch fermentation. This study offers a possible approach for the industrial bioproduction of l-ABA and paves a way for the industrialization of other nonnatural amino acids.

## Methods

### Strains, media, and growth conditions

The bacterial strains used in this study were listed in Table 1. *E. coli* DH5α was used as host for the recombinant plasmid. *E. coli* W3110 and its derivatives were applied to produce l-ABA.

TPM medium was used to monitor the production of l-ABA, during the growth of cells at 35 °C. TPM medium contains per liter: glucose, 50 g; yeast extract, 6 g; MgSO_4_·7H_2_O, 2 g; KH_2_PO_4_, 4 g; (NH_4_)_2_SO_4_, 14 g; betaine, 1 g; l-methionine, 0.149 g; l-lysine, 0.164 g; trace metal solution, 5 mL and CaCO_3_, 30 g. The trace metal solution contains per liter: FeSO_4_·7H_2_O, 10 g; CaCl_2_, 1.35 g; ZnSO_4_·7H_2_O, 2.25 g; MnSO_4_·4H_2_O, 0.5 g; CuSO_4_·5H_2_O, 1 g; (NH_4_)_6_Mo_7_O_24_·4H_2_O, 0.106 g; Na_2_B_4_O_7_·10H_2_O, 0.23 g; 35% HCl, 10 mL [21]. The medium was adjusted to pH 7.0 by KOH. Ampicillin (100 mg/L; Sangon, Shanghai, China), kanamycin (50 mg/L; Solarbio, Beijing, China), and isopropyl-β-D-thio-galactopyranoside (IPTG; 100 µM; Sangon, Shanghai, China) were added when necessary.

For precultivation of *E. coli* W3110 and its derivatives, a single clone was grown in 5 mL Luria–Bertani (LB) medium. After incubation for 10 h, the seed culture was inoculated into 500 mL shake flask containing 30 mL cultivation media. Cells were grown at 35 °C and 100 mg/L ampicillin was supplemented when needed. For l-methionine auxotrophic and l-lysine auxotrophic mutants, l-methionine and l-lysine with final concentrations of 0.149 g/L and 0.164 g/L were added, respectively in fermentation broth to fairly compare their titer with others trains [32]. A final concentration of 0.1 mM IPTG was added to the medium for gene induction when the optical density at 600 nm (OD_600_) reached of 0.4–0.6. Fermentation of strains were conducted simultaneously under the same culture conditions for at least three times.

### Construction of co-expression plasmids

All plasmids used in this study were listed in Table 2. The primers used for gene amplification and recombinant plasmid construction were listed in Additional file 1: Table S1. In general, genes including *ilvA* encoding feedback-resistant threonine dehydratase and *leuDH* encoding l-leucine dehydrogenase from *Thermoactinomyces intermedius* were overexpressed in a plasmid-based manner. The procedures of recombinant plasmid construction were described in detail shown in Additional file 1: Figure S1.

### Genomic engineering: promoter replacement, site-directed mutagenesis and chromosomal gene knock-out

Donor dsDNA with 500-bp homologous arms on each side was designed based on the sequence of the gene cluster and the location of sgRNA. Two homologous arms and the Trc promoter were separately amplified and fused together by overlap-extension PCR. The PCR products were purified by gel extraction prior to electroporation. Electrocompetent cells were prepared according to a previous report [33]. A single colony was transferred into 5 mL of LB medium containing 50 mg/L kanamycin and 10 mM l-arabinose (Aladdin, Shanghai, China), and was grown at 30 °C overnight. An aliquot consisted of 100 µL resulting preculture was transferred into 50 mL of LB medium containing 50 mg/L kanamycin and 10 mM l-arabinose, and was grown at 30 °C to an OD_600_ value of 0.4–0.6. The cultures were chilled in ice-water slurry for 15 min, harvested by centrifugation at 4000×*g* for 10 min, and washed twice with ice-cold sterile ddH_2_O. Subsequently, 200 µL of ice-cold sterile glycerol (10%, v/v) was used to re-suspend the cells, and the glycerol suspension was separated into 100 µL aliquots for each reaction. Donor dsDNA (400 ng) and the corresponding pTarget plasmid (100 ng) were added to each electroporation reaction. A Bio-Rad MicroPulser (Bio-Rad, Hercules, CA, USA) was used for electroporation (0.1 cm cuvette, 1.8 kV). 1 mL of pre-chilled LB medium was added to the cuvette and the resulting cell suspension was transferred into a tube within 1 min. The culture was then regenerated at 30 °C for 2 h prior to plating. Positive colonies were transferred into LB medium containing 0.5 mM IPTG and cultivated at 30 °C for 8–10 h to eliminate the pTarget plasmid. The pCas plasmid was cured by cultivating at 37 °C overnight. The cultures after plasmid curing were streaked, and the colonies were tested for kanamycin (50 mg/L) and spectinomycin (50 mg/L; Sangon, Shanghai, China) sensitivity, and were confirmed by sequencing [19, 34].

### Fermentation

For fed-batch fermentation, a 150 mL seed cultured in LB medium for 12 h was inoculated into a 5 L agitated bioreactor (Shanghai Baoxing biological equipment Engineering Co. Ltd, China) with 3 L TPM medium equipped with dissolved oxygen, pH and temperature probes. Cells were incubated at 35 °C with agitation at 500 rpm and 2 L/min external air flow. Ammonia or phosphate was automatically fed into the broth to keep the pH at 7.0. When the glucose concentration in the broth was less than 5 g/L, 100 mL of feed solution (500 g/L glucose, 14 g/L (NH_4_)_2_SO_4_, 12.5 g/L KH_2_PO_4_, 3 g/L l-methionine and 4.4 g/L l-lysine) was injected into the broth to raise the residual glucose concentration to around 20 g/L [21, 35].

### Analytical methods

The cell concentration was monitored by measuring the absorbance at 600 nm which was then converted to Dry Cell Weight (DCW) by a calibration curve [36]. The fermentation supernatants were filtered through a 0.22 µm syringe filter (Nylon66; Jinteng, Tianjin, China) and used for determination of residual glucose, amino acids and organic acids. The concentration of 2-KB in the culture was determined by high pressure liquid chromatography (HPLC, Waters, Milford, MA, USA) using an Aminex HPX-87H column (7.8 × 300 mm, Bio-Rad, Hercules, CA, USA) under the following conditions: mobile phase 5 mM H_2_SO_4_; flow rate 0.5 mL/min; column temperature 30 °C; UV absorption 215 nm [31]. The residual concentration of glucose in the media was measured using a glucose analyzer (YSI model 2300, Xylem Inc., Rye Brook, NY, USA) [37] and the amino acids were determined using an amino acid analyzer (SYKAMS-433D, SYKAM, Munich, Germany). The l-Thr and l-ABA were derivatized with DNFB/Acetonitrile (DNFB = 1%, acetonitrile = 99%) and analyzed by HPLC using a LC-18DB column (5 µm, 4.6 × 250 mm, Agilent, Beijing, China). Derivatization method: 100 μL of the fermentation supernatants diluted 10 times with ddH_2_O were added to a 1.5 mL centrifuge tube, and then 50 μL of DNFB/Acetonitrile buffer and 100 μL of 0.5 mol/L NaHCO_3_ buffer were added. The reaction solution was placed in a dark water bath at 60 °C for 1 h. After the reaction was completed, 750 μL of 0.2 mol/L phosphate buffer at pH 7.0 was added. Finally, the reaction solution was filtered through a 0.22 µm syringe filter. The gradient elution profile, at 1 mL/min, was as follows: 16% A and 84% B at 0–0.18 min, 30% A and 70% B at 0.18–2.4 min, 34% A and 66% B at 2.4–4.2 min, 43% A and 57% B at 4.2–7.2 min, 55% A and 45% B at 7.2–13.3 min, 55% A and 45% B at 13.3–15 min, 98% A and 2% B at 15–20.4 min, 16% A and 84% B at 20.4–21.3 min, 16% A and 84% B at 21.3–30 min (A = 50% acetonitrile; B = 4.1 g/L sodium acetate, pH adjustment to the value of 6.4 with acetic acid); column temperature 33 °C; UV absorption at 360 nm. The retention time of the main peak of the solution should be consistent with that of the reference solution. The undiluted medium and fermentation broth of *E. coli* W3110/pTrc99A were used as negative controls. Amino acid standards were purchased from Sykam (Amino Acid Calibration Solution H, Sykam, Germany).

### Statistical analysis

Unless otherwise specified, all experiments in this study were performed in triplicates. An analysis of variance (ANOVA) was carried out using the SAS program version 8.1 (SAS Institute Inc., Cary, NC, USA). The least significant difference (LSD) was computed at *p* < 0.05. All figures were drawn using Origin software version 8.5 (Origin Lab Corp., Northampton, MA, USA). Error bars denote standard deviation of the mean.

## Additional file

**Additional file 1: Table S1.** Primers for donor DNA amplification, pTarget construction and positive colony validation. **Figure S1.** Structures of plasmids used in this study. Detailed description on constructions of these plasmids is shown in the section.

## Abbreviations

l-ABA: l-2-aminobutyric acid
2-KB: 2-ketobutyrate
DCW: dry cell weight
IPTG: isopropyl β-d-1-thiogalactopyranoside
l-Thr: l-threonine
eGFP: enhanced green fluorescent protein
