# Supplementary material for: Fermentative production of the unnatural amino acid l-2-aminobutyric acid based on metabolic engineering
Source: Microb Cell Fact. 2019 Feb 28;18:43. doi: 10.1186/s12934-019-1095-z (PMC6393993; doi:10.1186/s12934-019-1095-z)
Supplement: Supplementary file 1 — Additional file 1: Table S1. Primers for donor DNA amplification, pTarget construction and positive colony validation. Figure S1. Structures of plasmids used in this study. Detailed description on constructions of these plasmids is shown in the section. [file 12934_2019_1095_MOESM1_ESM.docx]

**Additional file**

**Fermentative production of the unnatural amino acid L-2-aminobutyric acid based on metabolic engineering**

Jian-Miao Xu, Jian-Qiang Li, Bo Zhang, Zhi-Qiang Liu*, Yu-Guo Zheng

*Key Laboratory of Bioorganic Synthesis of Zhejiang Province, College of Biotechnology and Bioengineering, Zhejiang University of Technology, Hangzhou 310014, China*

***Corresponding author:**

Fax: +86-571-88320630; Tel: +86-571-88320379; E-mail: microliu@zjut.edu.cn

**Table S1.** Primers for donor DNA amplification, pTarget construction and positive colony validation.

| **Primer** | **5’-3’ Sequence** |
| --- | --- |
| *ilvA*-F1  *ilvA*-R1  *leuDH*-F1  *leuDH*-R1  *ilvA-*up  *ilvA-*down  ZF-2  ZR-2  *BleuDH*-F1  *BleuDH*-R1  Trc-F2  Trc-R2  Gap-F2  Gap-R2  Pbs-F2  Pbs-R2  ZF-1  ZR-1  Gap-F1  Gap-R1  Pbs-F1  Pbs-R1  pTarget-*rhtA* F  pTarget-*rhtA*R  donor-*rhtA*1 F  donor-*rhtA*1 R  donor-*rhtA*2 F  donor-*rhtA*2 R  pTarget-*ilvIH* F  pTarget-*ilvIH* R  donor-*ilvIH*1 F  donor-*ilvIH*1 R  donor-*ilvIH*2 F  donor-*ilvIH*2R | CAGACCATGGAATTCGAGCTCAAGGAGGTTTCTTATGGCTGACTCGCAACCCC  CAGGTCGACTCTAGAGGATCCCTAACCCGCCAAAAAGAACCTGAAC  GCGGGCTAAGGATCCTCTAGAAAGGAGGTTTCTTATGGGTAAAATCTTCGAC  CATCCGCCAAAACAGCCAAGCTTTTATTTGTTGTTGAAGTTG  CAGTTGGCAGAATTTGAGGGCGCTGCCTT  CTGCCAACTGCTTGGCGGGTTCTCGGTCAC  AAGCTTGGCTGTTTTGGCGGATG  TCTAGAGGATCCTTAGCCCGC  GCGGGTTAGGGATCCTCTAGAAAGGAGGTTTCTTATGACCCTGGAAATTTTTG  CATCCGCCAAAACAGCCAAGCTTTTAACGGCGGCTAATAATATC  CTTTTTGGCGGGTTAGGGATCCTTGACAATTAATCATCCGGC  CCCATAAGAAACCTCCTTTCTAGATTGTTATCCGCTCACAATTC  CTTTTTGGCGGGTTAGGGATCCGAGGCGAGTCAGTCGCGTAATGC  AACCTCCTTTCTAGAGGATCCTTAATTAAGATCTATATTCCACCAGCTATTTGTTAG  CTTTTTGGCGGGTTAGGGATCCGGCGCGCCCCTCCTTGACACTG  AACCTCCTTTCTAGAGGATCCGGAATTGTTATCCGCTCACAATTCCAAGCTTGGGTAGAATATTAAG  TGTGGAATTGTGAGCGGATAAC  CAGCTCATTTCAGAATATTTGCCAG  CAAATATTCTGAAATGAGCTGGAGGCGAGTCAGTCGCGTAATG  GTTATCCGCTCACAATTCCACATTAATTAAGATCTATATTCCACCAGC  CAAATATTCTGAAATGAGCTGGGCGCGCCCCTCCTTGACACTGAATTTAG  GTTATCCGCTCACAATTCCACAAAGCTTGGGTAGAATATTAAG  TAATACTAGTAGTCATATTGCTCGTTGCCAGTTTTAGAGCTAGAAATAGC  GCTCTAAAACTGGCAACGAGCAATATGACTACTAGTATTATACCTAGGAC  CGGTGCTTTTTTTGAATTCTCTAGACGGGCTGTTGTCTTCTTCAT  AAATGTAAATTCTTTCTCCCACAAATATCT  GGGAGAAAGAATTTACATTTCTGCATGGTT  GGGTAATAGATCTAAGCTTCTGCAGTGATAACTTGAGTGGTCCCC  TAATACTAGTCAGGGCGTTAAACAAGTATTGTTTTAGAGCTAGAAATAGC  GCTCTAAAACAATACTTGTTTAACGCCCTGACTAGTATTATACCTAGGAC  CGGTGCTTTTTTTGAATTCTCTAGAGACACGGTAGATAAGCAAGC  CGGCGCATAAGGCCTGCCTCACTGTTTGAC  GAGGCAGGCCTTATGCGCCGGATATTATCA  GGGTAATAGATCTAAGCTTCTGCAGCGCAAAAAAAGCCCAACGTG |

**Fig. S1** Structures of plasmids used in this study. Detailed description on constructions of these plasmids is shown in the section.

The gene sequence of *ilvA* was amplified from *E. coli* W3110 using primers *ilvA*-F1 and *ilvA*-R1 with homologous arms. Site-directed mutagenesis (1054^th^ T with G, 1055^th^ T with C, 1084^th^ C with T, 1085^th^ G with T and 1086^th^ T with C) were performed to obtain *ilvA** using *ilvA*-up and *ilvA*-down as primers, after which the *ilvA** fragment was inserted between the *Sac* I and *BamH* I restriction sites of vector pTrc99A to obtain recombinant plasmid pTrc-*ilvA**. The sequence of *leuDH* from *T. intermedius* was amplified using primers *leuDH*-F1 and *leuDH*-R1, whereby an additional SD sequence (AAGGAG) followed by a spacing sequence (ATATAC) before the start codon of *leuDH* was designed on the primer *leuDH*-F1. The amplified fragment was then inserted into pTrc-*ilvA** between the *Xba* I and *Hind* III restriction sites to construct pTrc-*ilvA**-*leuDH*. A fragment containing a trc promoter was amplified using Trc-F2 and Trc-R2, and then inserted into pTrc-*ilvA**-*leuDH* between the *BamH* I and *Xba* I restriction sites to obtain pTrc-*ilvA**-Trc-*leuDH*. Other recombinant plasmids were constructed in the same way.
